# Identification of Anti-SARS-CoV-2 Compounds from Food Using QSAR-Based Virtual Screening, Molecular Docking, and Molecular Dynamics Simulation Analysis

**DOI:** 10.3390/ph14040357

**Published:** 2021-04-13

**Authors:** Magdi E. A. Zaki, Sami A. Al-Hussain, Vijay H. Masand, Siddhartha Akasapu, Sumit O. Bajaj, Nahed N. E. El-Sayed, Arabinda Ghosh, Israa Lewaa

**Affiliations:** 1 Department of Chemistry, Faculty of Science, Imam Mohammad Ibn Saud Islamic University (IMSIU), Riyadh 13318, Saudi Arabia; sahussain@imamu.edu.sa; 2 Department of Chemistry, Vidya Bharati Mahavidyalaya, Amravati, Maharashtra 444 602, India; 3 Corden Pharma, Boulder, CO 80301, USA; asidhu09@gmail.com (S.A.); sumitbajaj16@gmail.com (S.O.B.); 4 Egyptian Drug Authority, Agouza, Giza 35521, Egypt; nnelsayed@gmail.com; 5 Microbiology Division, Department of Botany, Gauhati University, Guwahati, Assam 781014, India; dra.ghosh@gauhati.ac.in; 6 Department of Business Administration, Faculty of Business Administration, Economics and Political Science, British University in Egypt, Cairo 11837, Egypt; Israa.lewaa@bue.edu.eg

**Keywords:** SARS-CoV, SARS-CoV-2, COVID-19, QSAR, molecular docking, QSAR-based virtual screening, machine learning

## Abstract

Due to the genetic similarity between SARS-CoV-2 and SARS-CoV, the present work endeavored to derive a balanced Quantitative Structure−Activity Relationship (QSAR) model, molecular docking, and molecular dynamics (MD) simulation studies to identify novel molecules having inhibitory potential against the main protease (Mpro) of SARS-CoV-2. The QSAR analysis developed on multivariate GA–MLR (Genetic Algorithm–Multilinear Regression) model with acceptable statistical performance (R^2^ = 0.898, Q^2^loo = 0.859, etc.). QSAR analysis attributed the good correlation with different types of atoms like non-ring Carbons and Nitrogens, amide Nitrogen, sp^2^-hybridized Carbons, etc. Thus, the QSAR model has a good balance of qualitative and quantitative requirements (balanced QSAR model) and satisfies the Organisation for Economic Co-operation and Development (OECD) guidelines. After that, a QSAR-based virtual screening of 26,467 food compounds and 360 heterocyclic variants of molecule **1** (benzotriazole–indole hybrid molecule) helped to identify promising hits. Furthermore, the molecular docking and molecular dynamics (MD) simulations of Mpro with molecule **1** recognized the structural motifs with significant stability. Molecular docking and QSAR provided consensus and complementary results. The validated analyses are capable of optimizing a drug/lead candidate for better inhibitory activity against the main protease of SARS-CoV-2.

## 1. Introduction

The current epidemic involving the coronavirus disease 2019 (COVID-19), caused by severe acute respiratory syndrome coronavirus 2 (SARS-CoV-2), has completely disturbed the global health system and the world economy. The first patient of COVID-19 was reported in Wuhan, China, in December, 2019 [1], and now the virus has reached 215 countries in the world [2]. In some countries, it is in stage one or stage two, but in some countries, it has reached stage three, i.e., community spread [2]. Due to its high spreading rate and mortality, WHO (World Health Organization) has declared it a Public Health Emergency of International Concern [2].

SARS-CoV-2 belongs to the family of Coronaviridae and the genus Betacoronavirus [3]. These are enveloped (enclosed) viruses having a single positive-stranded RNA genome (~26–32 kb in length) [4]. The name “Corona” has been given to them due to the crown-like presence of a fringe of large and bulbous surface projections when observed under the microscope [5]. SARS-CoV-2 is the seventh member [6] from this family to infect the humans, other mammals, and birds [7]. Coronavirus infections mainly cause respiratory tract infections, and some of them are responsible for severe infectivity and damage to public health, e.g., in 2002, severe acute respiratory syndrome (SARS); in 2012, Middle East respiratory syndrome (MERS); and now, COVID-19 [7]. It has been established that SARS-CoV-2 has ~80% similarity in genetic sequence with other coronaviruses, especially with SARS-CoV [8]. It can easily spread through contact or respiratory droplets. Most of the infected patients are asymptomatic, which creates a real challenge to search such infected persons and control further spreading of COVID-19. Therefore, travel restrictions, social distancing, personal hygiene, and quarantine measures have been implemented by many countries to curb further spread of this virus [9].

As this global pandemic has recently appeared and developing a new medicine for a disease usually takes a lot of time, to date, there is no side-effect-free vaccine or drug available for the prevention or the treatment of COVID-19. However, certain active pharmaceuticals, developed for other diseases, such as anti-malarial (Chloroquine and Hydroxychloroquine), anti-Ebola (Remdesivir, etc.), and vaccines from Moderna, Pfizer, etc., have gained emergency use authorization from drug regulatory agencies in many countries for management of critical COVID-19 infections [10,11,12]. The appearance of new variants of SARS-CoV-2 with higher rate of spread is a concern [13]. In an another approach, convalescent plasma containing antibodies from COVID-19 recovered some of the patients; oxygenation [14] and ventilation are also among the possible treatment strategies. However, these therapies are not ultimately effective. Therefore, the scientific community is still working on the development of specific COVID-19 drugs, and studies are going on in several directions for different type of patients. In this regard, a good number of researchers employed molecular docking and Quantitative Structure−Activity Relationship (QSAR) for virtual screening to identify novel hits for SARS-CoV-2 [11,15,16].

For the fast and cheaper development of medicines treating SARS-CoV-2, drug repositioning strategy is proposed. This approach is based on exploring the anti-SARS-CoV-2 potential of leverage molecules earlier reported to have inhibitory activity against genetically related viruses such as SARS-CoV.

A high semblance of SARS-CoV-2 with SARS-CoV is a primary hope to develop a safer and cheaper drug candidate. That is, the existing knowledge about SARS-CoV, a high similarity of SARS-CoV-2 with SARS-CoV, and a compound previously tested for SARS-CoV is a viable candidate to develop a therapeutic drug. It is rational to believe that a SARS-CoV inhibitor could also be equipotent against SARS-CoV-2. In other words, a cheaper, time-saving, feasible strategy is to leverage molecules earlier reported to have inhibitory activity against similar viruses such as SARS-CoV.

The e-Chemistry approach [17] (easy, efficient, economical, and eco-friendly) is to be followed to make the process of drug designing cheaper, more result oriented, and less time-consuming, with less trials and errors. The field of drug design has witnessed the rise of Computer Aided Drug Designing (CADD) as a fast, efficient, and cheaper tool for lead/drug optimization [18] with the advent of computers and allied resources. The CADD analysis is useful to identify the pharmacophoric features, predict the bio-chemical profile of a compound before its actual synthesis and bio-screening, and to understand the mechanism of action of molecules. The success of QSAR, molecular docking, and pharmacophore modeling has led to their regular utilization, to develop effective strategies to optimize a lead/drug candidate.

The field of QSAR has emerged as an attractive methodology to identify the important structural features and predict the desired activity/property, using a series of molecules. For a QSAR analysis to be successful, it should have a balance of mechanistic (Descriptive/Qualitative) and predictive (Quantitative) approaches [19,20,21,22]. In Qualitative QSAR, the main impetus is to understand the influence of molecular structure on the mode of action or the biological mechanism of studied chemicals, whereas, in Quantitative QSAR, the main focus is on statistical validation of developed QSAR models, to verify their performance, i.e., statistical robustness and predictive ability for new chemicals [23].

Generally, researchers use molecular docking based HTS (high-throughput screening) for identifying hits, but its hit rate ranges between 0.1 and 0.01% [24]. Therefore, the overall cost of drug discovery substantially increases as most of the tested compounds are regularly found to be false positives toward the desired bioactivity [25]. On the other hand, QSAR-based virtual screening (VS) has been used to identify hits for drug designing, and its typical hit rates ranges from 1 to 40% [24]. This higher success rate not only highlights the advantages of using QSAR-based VS, it also brings QSAR modeling in tune with the final needs of experimental medicinal chemists to discover bioactive compounds. In other words, this approach extends the focus of QSAR modeling from accomplishing statistically significant training set models toward utilizing the validated models to rank molecules for subsequent biological evaluation. In the present work, a database of food compounds and a good number of heterocyclic variants of most active compound 1 were used to perform the QSAR-based VS. An obvious reason for choosing the food compounds is the innate ability of many foods compounds to have immune stimulatory effects without toxicity [26,27].

Molecular docking is a contemporary structure-based drug design approach, which involves simulations of interactions between a lead/drug molecule with a target enzyme. It provides an in-depth knowledge and understanding of binding patterns to identify the important structural features of a small molecule that are responsible for its binding with the enzyme [28].

Thus, a concomitant use of QSARs and molecular docking not only provides consensus and complementary information about prominent structural features but it is useful also to predict the bio-activity of a compound before its wet lab synthesis and testing. Therefore, in the present work, we employed QSARs, QSAR-based virtual screening, and molecular docking analyses to achieve the desired goals.

## 2. Results

In the present work, the QSAR analysis and molecular docking were performed to identify important structural features. The QSAR model was build using easily interpretable molecular descriptors to correlate them with structural features. The five-parametric GA–MLR model has good external predictive ability with the presence of easily understandable molecular descriptors along with interpretation in terms of structural features. Even though, in the present analysis, a straight evaluation of Ki values of the molecules of the dataset was performed to explain the effect of a specific descriptor, it is important to note that the combined or converse effect of unknown factors or other molecular descriptors could have a substantial effect on the Ki value of a molecule.

### GA–MLR QSAR Model

pKi = 4.618 (± 0.415) + 2.774 (± 0.475) * fnotringCamdN3B + 0.762 (± 0.135) * aroN_sp3O_4B + 0.035 (± 0.021) * fringClipo5B + 0.962 (± 0.262) * flipoplaN2B −0.279 (± 0.089) * com_sp2O_5A

*R*^2^*_tr_* = 0.898, *R*^2^*_adj._* = 0.886, *LOF* = 0.291, *RMSE_tr_* = 0.432, *MAE_tr_* = 0.349, *CCC_tr_* = 0.947, *s* = 0.460, *F* = 77.120, *R*^2^*_cv_* (*Q*^2^*loo*) = 0.859, *RMSE_cv_* = 0.507, *MAE_cv_* = 0.402, *CCC_cv_* = 0.926, *Q*^2^*_LMO_* = 0.848, *R*^2^*_Yscr_* = 0.102, *Q*^2^*_Yscr_* = −0.164, *RMSE_ex_* = 0.648, *MAE_ex_* = 0.537, *R*^2^*_ex_* = 0.799, *Q*^2^*−F^1^* = 0.795, *Q*^2^−*F*^2^ = 0.795, *Q*^2^−*F*^3^ = 0.769, *CCC_ex_* = 0.893

**Figure 1:**
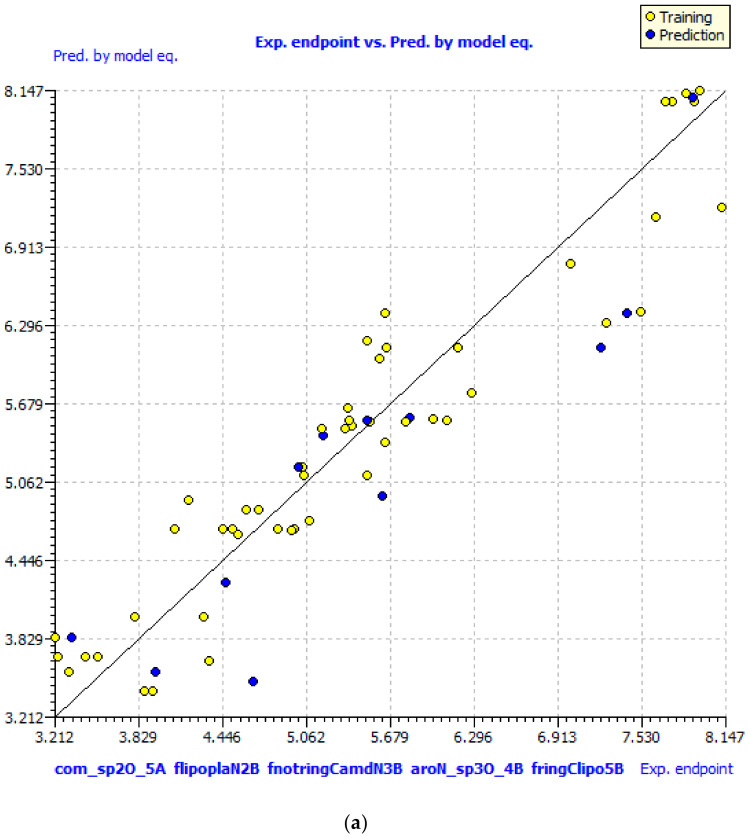

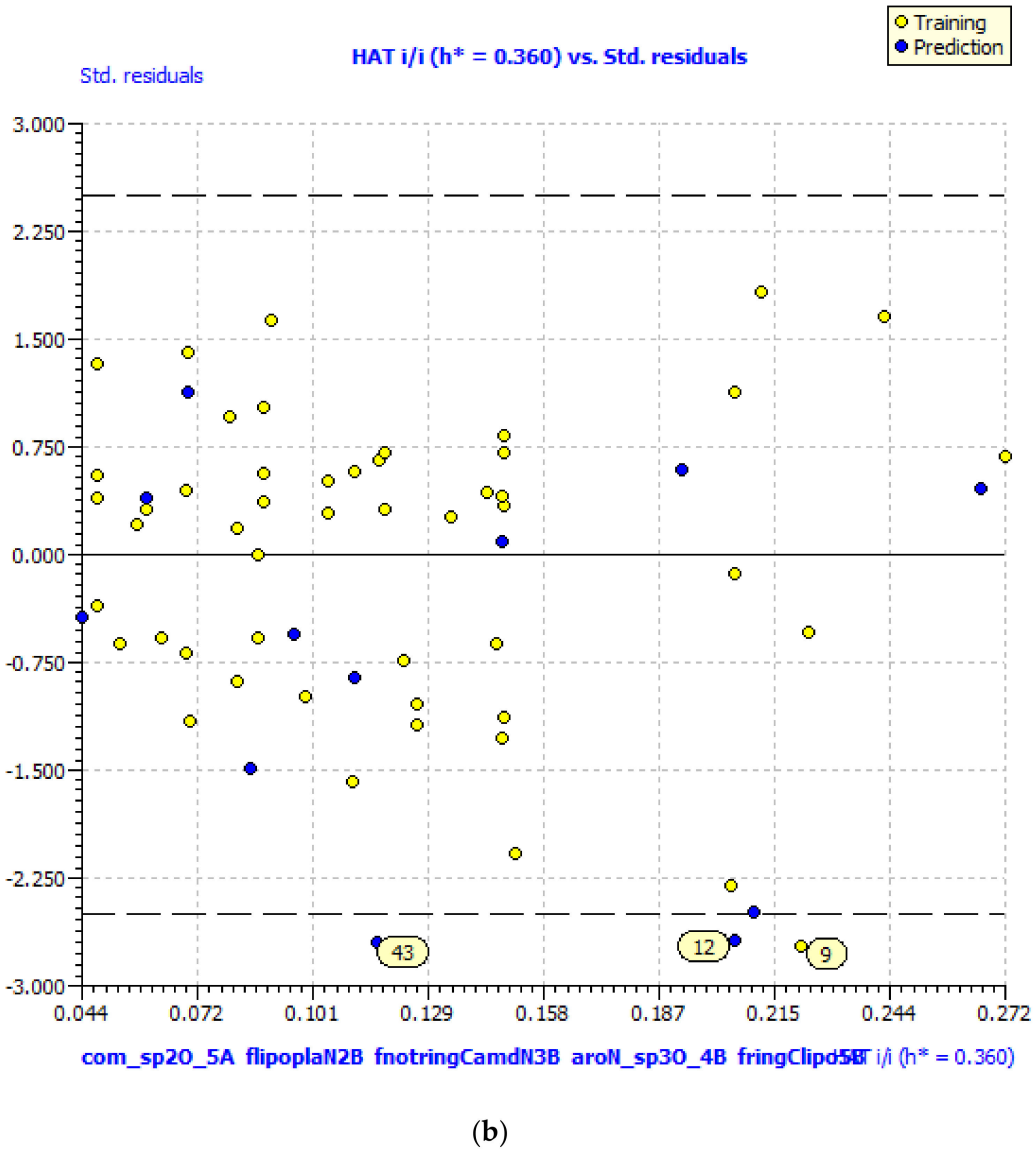
Different graphs associated with the developed Quantitative Structure−Activity Relationship (QSAR) model: (**a**) experimental vs. predicted pKi and (**b**) Williams plot to assess applicability domain of model. (molecules out of applicability domain have been shown with their serial numbers).

The different graphs associated with the developed QSAR model, i.e., experimental vs. predicted pKi and the Williams plot to assess applicability domain of model, are depicted in Figure 1. The developed QSAR model satisfies the recommended threshold values for a good number of statistical parameters (see Supplementary Materials for formulae and meaning of statistical parameters) [17,20,21,22,29,30,31,32]. The close values of *R*^2^*_tr_* (coefficient of determination), *R*^2^*_adj._* (adjusted coefficient of determination), LOF (lack of fit), and *R*^2^*_cv_* (*Q*^2^*loo*) (cross-validated coefficient of determination for leave-one-out) indicate that the model comprises optimum number of variables and free from over-fitting. The high value of *Q*^2^*_LMO_* (cross-validated coefficient of determination for leave-many-out) confirms the acceptable internal validation of a model. The low value of *R*^2^*_Yscr_* and *Q*^2^*_Yscr_* (Y-scrambling related parameters) suggests that the developed model is free from chance correlations. The high value of *R*^2^*_ex_* (external coefficient of determination), *Q*^2^*−F^n^* and *CCC_ex_* (Concordance Correlation Coefficient) vindicate the acceptable external predictive ability. The *F* (Fischer F-ratio) value vindicates the high statistical significance of the model. In addition, an analysis of the correlation matrix (see Supplementary Materials Table S3) indicates that the molecular descriptors have low correlation with each other.

## 3. Discussion

**fnotringCamdN3B**: The molecular descriptor fnotringCamdN3B signifies the frequency of occurrence of amide Nitrogen atoms exactly at three bonds from the non-ring Carbon atoms. If the same amide Nitrogen atom was simultaneously present at one or two bonds from any other non-ring Carbon atom, then it was excluded during the calculation of fnotringCamdN3B. This molecular descriptor has positive coefficient in the developed model, therefore increasing its value could lead to a better Ki value. As the amide Nitrogen is always a constituent of amide group, therefore it is reasonable to believe that this molecular descriptor highlights the importance of amide groups. A simple analysis reveals that the peptidomimetic molecules (**10**–**13**, **15**, **20**, and **21**) of the present dataset contain such a combination of amide Nitrogen and non-ring Carbon atoms as pyrrolidin-2-one ring. Konno et al., and Regnier et al. [33,34] have also reported that the pyrrolidin-2-one ring occupies the S1 pocket. This indicates the crucial role of pyrrolidin-2-one ring for potent inhibitory activity against SARS.

**aroN_sp3O_4B**: The molecular descriptor aroN_sp3O_4B points out the presence of aromatic Nitrogen atoms within foru bonds from sp^3^-hybridized Oxygen atoms. It is another molecular descriptor with positive coefficient in the developed model, therefore increasing the number of such combinations could augment the Ki value. Only eight molecules, which are also the top eight active candidates, possess a higher value of this molecular descriptor. These eight molecules are benzotriazole derivatives. This could be a reason for better Ki value for benzotriazole derivatives. The docking pose for molecule **1** supports the importance of aromatic Nitrogen atoms (see the docking Section 3.2). The three Nitrogen atoms of the Benzotriazole ring are present within four bonds from the sp^3^-hybridized Oxygen atom.

**fringClipo5B**: A molecular descriptor which increases the Ki value, due to positive coefficient in the developed QSAR model, is fringClipo5B. It represents the frequency of occurrence of lipophilic atoms exactly at five bonds from ring Carbon atoms. If the same lipophilic atom is simultaneously present at four or less bonds from any other ring Carbon atom, then it was excluded during the calculation of fringClipo5B. Thus, this descriptor highlights the importance of ring Carbon atoms and lipophilic atoms. Another molecular descriptor which indicates the importance of lipophilic atoms is **flipoplaN2B**. It stands for the frequency of occurrence of planer Nitrogen atoms exactly at two bonds from the lipophilic atoms. If the same planer Nitrogen atom is simultaneously present at one bond from any other lipophilic atom, then it was excluded during the calculation of flipoplaN2B. These two molecular descriptors indicate that specific combinations of lipophilic atoms with ring Carbon atoms and planer Nitrogen atoms could be useful to increase the Ki value for a molecule.

**com_sp2O_5A:** The molecular descriptor com_sp2O_5A represents the total number of sp^2^-hybridized Oxygen atoms present within 5Å from the center of mass of the molecule. It has a negative coefficient in the developed QSAR model; therefore, increasing the number of such Oxygen atoms could result in decreased Ki value for a molecule for main protease. A poor Ki value for molecule **43** (4.68 M), **49** (4.34 M), **53** (3.95 M), **54** (3.95 M), **55** (3.87 M), and **60** (3.32 M) could be attributed to high frequency of occurrence of such Oxygen atoms within 5Å from center of mass of molecules (com_sp2O_5A = 5). Interestingly, all these molecules are peptidomimetic derivatives. There are five peptidomimetic molecules present in the top 15 molecules from the present dataset, which contain two to four such Oxygen atoms within 5Å from center of mass of molecules. Therefore, it is reasonable to say that the value of **com_sp2O_5A** should be less than five to a have good Ki value.

In the present QSAR analysis, the constituent molecular descriptors of the GA–MLR QSAR model have provided salient and hidden information about the structural features related to diverse set of molecules tested for their activity for the main protease (Mpro). It is essential to understand that no single molecular descriptor can completely explain the observed distribution of Ki for such a diverse set of molecules. That is, the performance of the developed QSAR model relies on the concomitant use of constituent molecular descriptors.

### 3.1. QSAR-Based Virtual Screening

The SMILES notations, calculated values of molecular descriptors, pKi and Ki for different variants of compound **1** and food compounds used for virtual screening are available in Supplementary Materials. For the sake of convenience, herein, we show the ten most active molecules from different variants of compound **1** and the ten most active molecules from the food database, as predicted by the developed QSAR model. From Table 1, it is clear that an increase in the number of aromatic Nitrogen atoms could lead to a better Ki value for a molecule.

### 3.2. Docking Analysis

**Figure 2:**
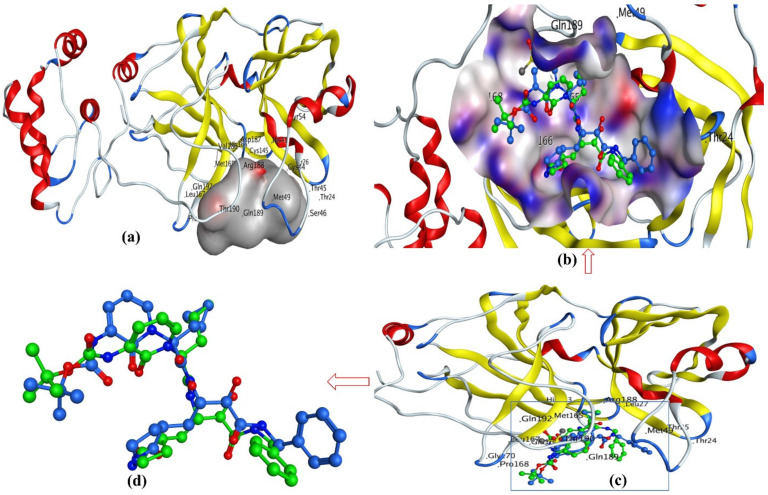
Docking and X-ray determined pose for **13b** to validate the docking protocol: (**a**) main protease (Mpro) with active site (gray-colored contour), (**b**) ligand **13b** in active site with surface, (**c**) without surface, and (**d**) comparison of docking pose for **13b** with X-ray determined pose.

Main protease (Mpro), also known as Nsp5 and 3CLpro, is a cysteine protease enzyme active in homodimer form only [17,35,36]. It is an essential enzyme for SARS-CoV-2, which participates in cleavage process of H-CoV polyproteins [6,17,35,36,37,38]. It has been established that it consists of three domains, domain I (residues 8–101), domain II (residues 102–184), and domain III (residues 201–303). A long loop (residues 185–200) connects domains II and III. The active site, which is highly conserved among all CoV’s Mpros and usually composed of four sites (S1′, S1, S2, and S4), is situated in the gap between domains I and II [6,17,35,36,37,38]. The catalytic dyad of Cys145 and His41 is an important feature of active site of this protein [6,17,35,36,37,38]. This protein is necessary for the processing of polyproteins and operates at 11 cleavage sites on the large polyprotein 1ab [8,35,39,40]. It exclusively breaks polypeptide sequences after a glutamine residue, whereas no human host-cell proteases are known to have such substrate specificity; therefore, an inhibitor of Mpro could be safe for humans [8,35,39,40]. In the current study, the dataset molecules and a known inhibitor 13b were docked inside the active site of Mpro. The X-ray resolution of **13b** is available, which was used to validate the docking protocol. Figure 2a shows the active site of the Mpro protein. The validation is performed by removing the crystal ligand **13b** from the active site and relocking it again. The alignment of Mpro with the crystal ligand **13b** and the redocked ligand is depicted in Figure 2b-d, which indicates that the docking protocol is acceptable (see Figure 2).

The docking scores for all the molecules are present in the Supplementary Materials. Table 2 contains the docking scores for the most active ten molecules. Even though molecule **1** is most active, molecule **10** has a better docking score. In fact, molecule **10** has the sixth best docking score (see Table 3). This means that the molecule **10** fits better inside the big active site of Mpro and fills it completely. This could be attributed to the larger size of molecule **10,** as compared to molecule **1**.

**Figure 3:**
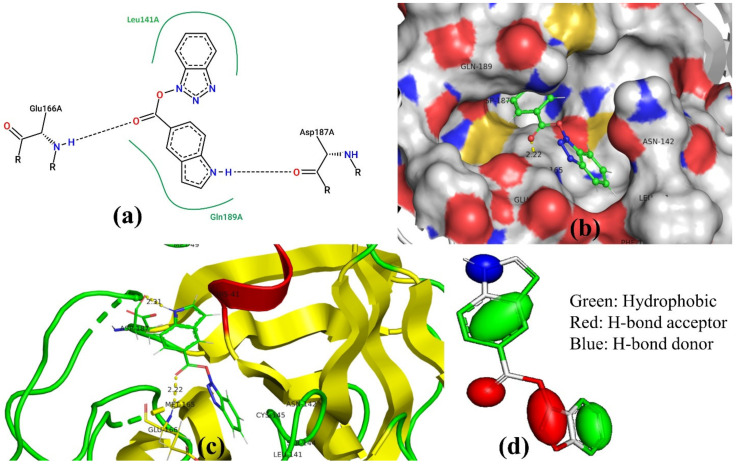
The docking pose for most active molecule **1** inside the active site of Mpro: (**a**) 2D representation of interactions, (**b**) with molecular surface, (**c**) without molecular surface, and (**d**) pharmacophore model.

For the sake of convenience, we present the docking pose and pharmacophore model for most active molecule **1** as a representative example (see Figure 3). In addition, the docking scores and the interacting amino acids for the least and most active molecules **1** and **62** are tabulated in Table 4.

### 3.3. Docking Pose for Most Active Molecule

From the docking pose, it appears that most active molecule **1** has occupied the S1 and S2 pockets of the active site. The benzene ring of benzotriazole moiety is present inside S1 pocket in proximity of Leu141 and responsible for lipophilic interactions. The polar triazole ring of benzotriazole moiety is oriented toward Met165 due to polar interactions. The importance of triazole has been also highlighted by the **aroN_sp3O_4B** molecular descriptor in QSAR analysis. The linker -COO- moiety is responsible for the establishment of H-bonding (2.22 Å) with Glu166. The benzene part of indole moiety is near to Gln189 due to lipophilic interactions. The same observation is vindicated by the presence of two molecular descriptors **flipoplaN2B** and **fringClipo5B**, which highlight the importance of lipophilic atoms in the newly developed QSAR model. The -NH- part of Indole ring is accountable for the H-bond formation with Asp187 (2.31 Å). Thus, QSAR and molecular docking analyses provide similar, as well as complementary, results.

The pharmacophore model generated by using the docking pose of most active compound **1** (see Figure 3c,d) indicates that a lipophilic region concomitant with a large H-bond acceptor region could avail in establishing useful interactions with the residues of S1 pocket, whereas a combination of large lipophilic region with H-bond donor is helpful to have key interactions with S2 pocket of Mpro.

### 3.4. MD Simulations and MMGBSA Binding Free-Energy Calculations

**Figure 4:**
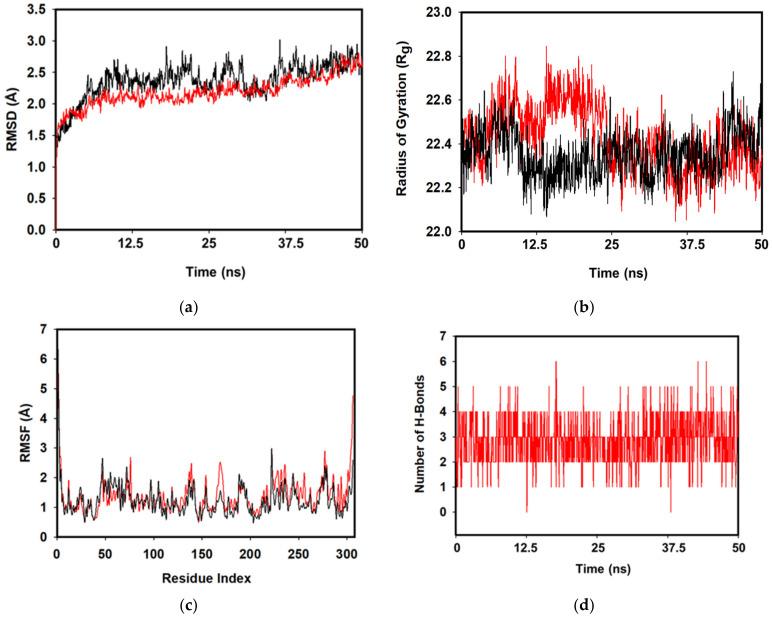

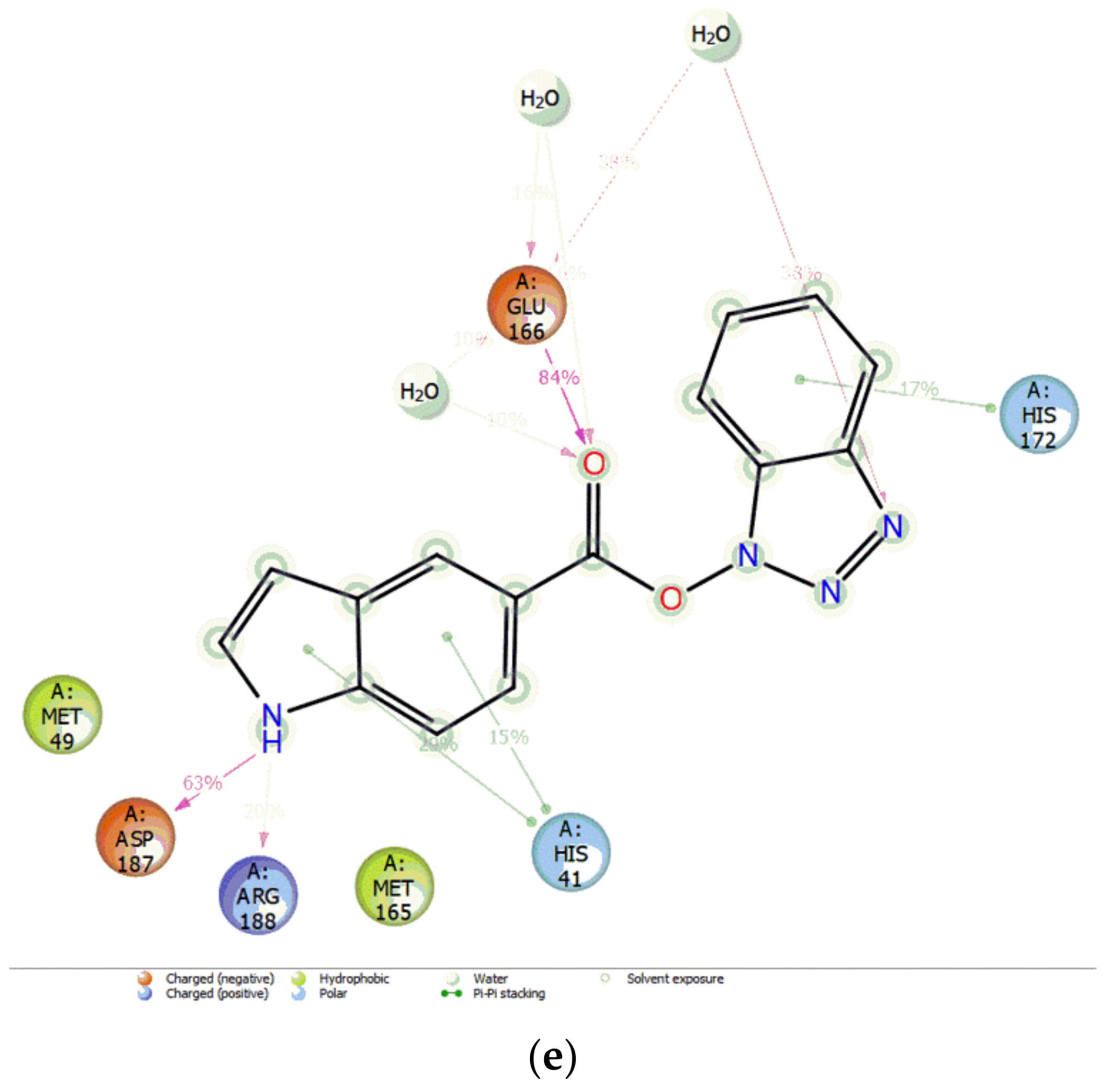
RMSD plots of (**a**) apo-Mpro (red) and **1** bound Mpro complex (black) implying overlapping vibrations and smooth interactions between protein and ligand. (**b**) Radius of gyration of apo-Mpro (red) and **1** bound Mpro complex (black), owing to their compactness after 50 ns simulation. (**c**) RMSF plots apo-Mpro (red) and **1**-Mpro (black) displaying least fluctuations throughout the 50 ns convergence during simulation. (**d**) H-bonds plot displaying the number of H-bonds formed during to total time scale of 50 ns of simulation. (**e**) Two-dimensional interaction plot of Mpro with **1** displaying the involvement of amino acids making varying interaction after 50 ns of simulation. The average H-bonds count displayed three numbers of bonds formation from the beginning to end of the simulation with the ligand **1** molecule (Figure 4d).

The final convergence and the stability of apo-Mpro and **1** bound Mpro were assessed in MD simulation studies. After 50 ns of convergence, apo-Mpro displayed RMSD of Cα backbone 2.2 Å. Initially, until 25 ns the backbone seemed to be stable but later from 25 to 50 ns of simulation the RMSD enumerated by average deviation 1.0 Å (Figure 4a, red). Whereas, RMSD plot of molecule **1** bound Mpro displayed the average deviation of 0.8 Å owing to its stable conformation over apo-Mpro protein. The interactions of protein and the molecule **1** displayed little RMSD differences (~0.8 Å) is acceptable conferred its stable conformation (Figure 4a, black). Radius of gyration is the indicator of size and compactness of the protein in the ligand bound state displayed in Figure 4b. The Rg plot of Cα-backbone of apo-Mpro (Figure 4b, red) has least compactness due to lowering of fluctuations 22.8 to 22.2 Å with an average of 22.35 Å form the beginning to end of the 50 ns simulation. On the other hand, Mpro bound complex with **1** displayed a constant gyration of the Cα backbone 22.4 Å with least deviations (Figure 4b, black). This signifies that the molecule **1** bound to Mpro complex is highly stable in comparison to the apo-Mpro. The RMSF plot displayed the stable conformation of each amino acid residues during of the simulation displaying fewer fluctuations in each amino acid positions in the complex of 1 bound Mpro (Figure 4c, black).

Mpro binding site for **1** displayed the major interactions, such as forming water bridges with negatively charged Glu166 and H-bond (Figure 4e). Asp187 and Arg 188 residues were also involved in H-bonding formation with the **1** molecule. Other residues include His41 and His172 involved in polar contacts, as well as pi-pi stacking, respectively. Therefore, the varying interactions played a critical role in stabilizing the whole complex (Figure 4e).

**Figure 5:**
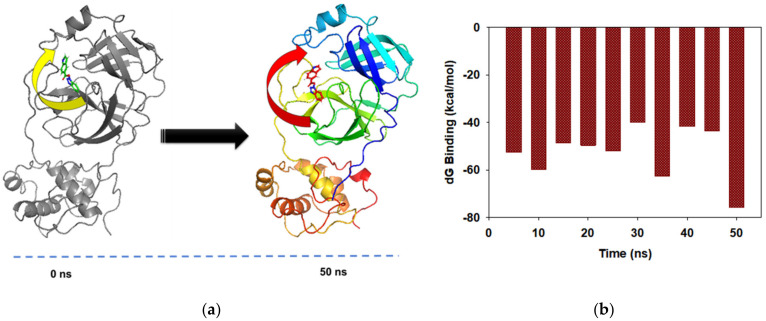
(**a**) Structural superimposition of first frame (0 ns) and last frame (50 ns) of **1** bound Mpro complex after simulation. The conformational change of secondary structure (arrow) observed at the **1** bound site and geometry of ligand displayed at 0 ns (yellow) and 50 ns (red). (**b**) Free-energy decomposition of binding energies at every 5.0 ns frame in MMGBSA calculations for 50 ns simulation.

Structural superimposition of initial and final frames of the **1** bound Mpro displayed mere changes at the binding site. The secondary fold of the protein at the converged state (at 50 ns) showed alteration at the α-Helical turn (Figure 5a, arrow) that facilitated better orientation of the molecule **1** geometry (red). Moreover, there was a tilt of the ligand (arrow, yellow = 0 ns; and arrow, red = 50 ns) during the course of simulation (Figure 5a). This signifies that the ligand was docked well at the binding site of the Mpro. The free energy change of ligand binding in the main protease Mpro of SARS-CoV-2 using MMGBSA calculations displayed the average (dG) binding = −52.54 ± 4 kcal/mol. The significantly high binding energy implied the higher affinity of molecule **1** with Mpro and perhaps opened the new arena for a novel inhibitor drug against SARS-CoV-2.

## 4. Materials and Methods

### 4.1. QSAR Analysis and Model Building

**Figure 6:**
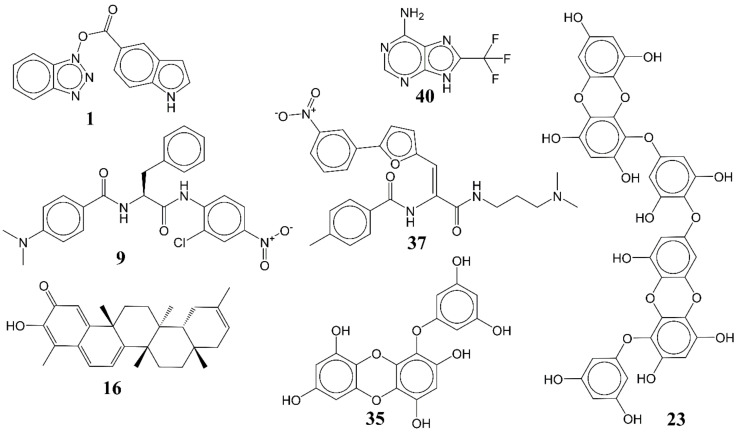
Representative examples of different classes of molecules used in the present work. (Bold numbers indicate serial number of molecules in the data set).

In the present work, a dataset of 351 molecules was downloaded from Binding database (https://www.bindingdb.org/bind/index.jsp (accessed on 28 September 2020)). The dataset contains diverse compounds, thus covering enough chemical space. Then, dataset curation involving the removal of salts, duplicates, and entries with ambiguous Ki values led to a reduced dataset of sixty-two compounds. However, the remaining sixty-two compounds have a wide range of activities against SARS-CoV (Ki = 7.5 to 614,000 nM), thus justifying the composition of the dataset. Afterward, prior to subsequent QSAR analysis, the reported Ki values were converted to pKi (pKi = −log_10_Ki). The Simplified Molecular-Input Line-Entry System (SMILES) notation for all selected molecules, along with their reported activity values Ki and pKi are present in the Supplementary Materials. Figure 6 contains the representative examples of different classes of molecules used in the present work. Moreover, Table 5 contains the SMILES notations, Ki (nM), and pKi (M) for the top five most active and least active molecules.

OpenBabel [41] ver. 2.4 was used to convert SMILES notation to 3D structures, using MMFF94 force field. The 3D structures were then used to calculate a myriad of molecular descriptors using “PyDescriptor” [42]. This led to a cluster of more than 15,000 molecular descriptors for each molecule. In the next step, QSARINS-2.2.4 [43,44] was used to remove constant, nearly constant and highly correlated (|R| > 0.90) molecular descriptors to avoid the inclusion of multi-collinear and redundant molecular descriptors in the process of development of a robust QSAR model. This significantly reduced the size of molecular descriptor pool (11,299 molecular descriptors), still containing a variety of 1D to 3D molecular descriptors, thus covering a broad descriptor space.

After that, GA–MLR (Genetic Algorithm–Multilinear Regression) [45] was used for subjective feature selection (SFS), using QSARINS-2.2.4 with default settings, except that the number of generations was set to 10,000. During SFS, the dataset was split randomly into the training set (80%) and the prediction (test or external) set (20%). The prediction set was not used during model building. It was only used for validation of the developed model. The heuristic search was limited to five variables to avoid over-fitting and enhance simplicity of the model. The GA–MLR module of QSARINS-2.2.4 uses Q^2^ as a fitness function. The developed QSAR model was subjected to thorough statistical validation (internal and external validation) according to Organisation for Economic Co-operation and Development (OECD) principles. Model with a high internal and external predictive ability has been reported. Further details about the QSAR model development are available in the literature [20,29,30,31].

### 4.2. QSAR-Based Virtual Screening

For QSAR-based virtual screening, the most active compound **1** served as a template to generate a good number of heterocyclic variants using RDKit (RDKit: open-source cheminformatics; http://www.rdkit.org (accessed on 28 September 2020)). This resulted in a pool of 360 different heterocyclic variants. Moreover, a database of 26,467 food compounds was downloaded from FooDB (http://foodb.ca/ (accessed on 28 September 2020)), followed by the application of rule of five; the removal of duplicates, salts, and metal derivatives led to a reduced dataset of 8453 molecules. Thus, 8813 (360 + 8453) molecules were used for QSAR-based VS. Prior to molecular descriptor calculations, the 3D structures of molecules were prepared in the same way as modeling set. Then, the molecular descriptors were calculated, and the properly validated five-parametric QSAR model was used to predict the biological property of novel compounds.

### 4.3. Molecular Docking Analysis

**Figure 7:**
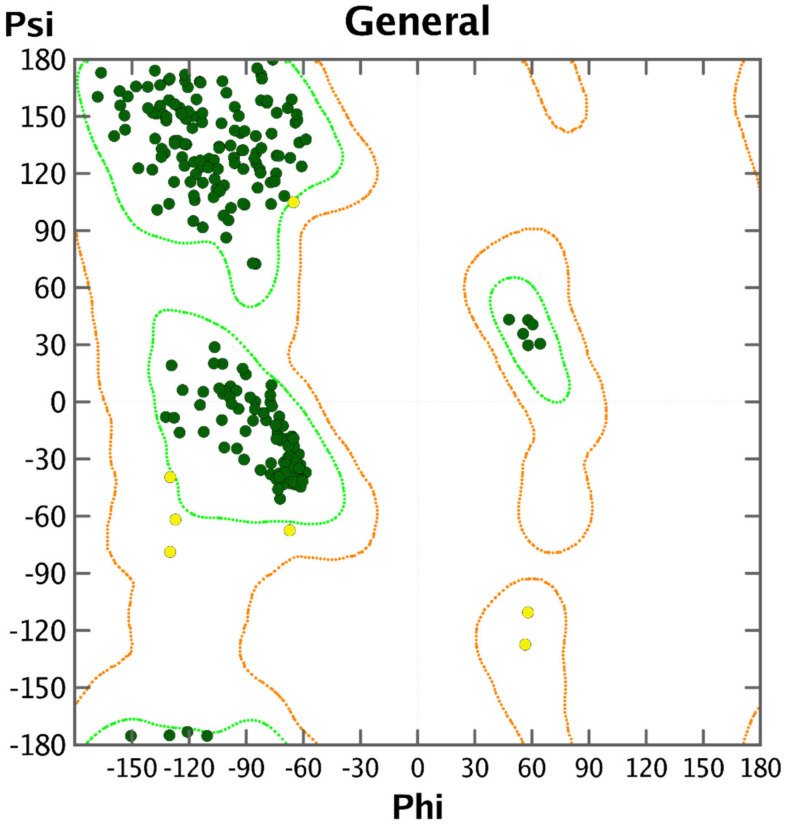
Ramachandran plot for main protease of SARS-CoV-2.

The pdb file for main protease was fetched from SWISS-MODEL (https://swissmodel.expasy.org/repository/species/ (accessed on 28 September 2020)). The pdb 6lu7 [37] was selected on the basis of X-ray resolution and completion of the sequence. Before actual docking simulations, the health of the protein was checked by plotting Ramachandran’s plot [46] (see Figure 7). The optimized protein is acceptable for docking analysis (see Figure 7). Prior to actual docking analysis, the native ligand N3 (a peptidomimetic inhibitor) was removed. All the compounds were docked in the active site, but for the sake of convenience, herein, the docking pose for most active molecule as a representative is depicted.

For the molecular docking analysis, the software NRGSuite [47] was used. This free software is available as a plugin for PyMOL (www.pymol.org (accessed on 28 September 2020)). It has the ability to detect the surface cavities in a protein and use them as target binding-sites for docking simulations with the help of FlexAID [48]. It uses genetic algorithm for conformational search, simulates ligand and side-chain flexibility and allows for the simulation of covalent docking. In the present work, flexible–rigid docking protocol was employed with following default settings to get optimum performance from NRGsuite: binding sites input method—spherical shape (diameter: 18Å); spacing of three dimensional grid—0.375Å; side chain flexibility—no; ligand flexibility—yes; ligand pose as reference—no; constraints—no; Hetero groups—included water molecules; van der Walls permeability—0.1; solvent types—no type; number of chromosomes—1000; number of generations—1000; fitness model—share; reproduction model—population boom; and number of top complexes—5 [22].

For validation of molecular docking, the molecule **13b**, a known peptidomimetic inhibitor of Mpro [35], was used to validate the docking protocol.

### 4.4. Molecular Dynamics and Binding Energy Calculations

Molecular Dynamics Simulation (MDS) studies were carried out in order to determine stability and convergence [49] of main protease main protease (Mpro) with and without molecule **1**. To set up the simulations, initially, the systems were built for complex **1**-Mpro and apo-Mrpo, respectively, in the system builder. For this purpose, Desmond 2018-4 was used to set up the initial parameters within explicit SPC water model orthorhombic box 4.0 × 4.0 × 4.0Å. The protease ligand complex and apo-Mpro were neutralized with NaCl salt by adding 0.15 M Na^+^ ions. The ASL module was used to select the specific residues of ligand and protein molecules for the better prepared systems, which were relaxed by using the Desmond default protocol of relaxation [50]. MDS run of 20 ns was set up at a constant temperature and constant pressure (NPT) for the final production run. The NPT ensemble was set up by using the Nosé–Hoover chain coupling scheme [51], at a temperature of 300 K. for final production and throughout the dynamics with relaxation time 1 ps. RESPA integrator was used to calculate the bonding interactions for a time step 2 fs [52]. All other parameters were associated in the settings followed as described elsewhere [53]. After the final production run, the simulation trajectories of main protease alone (apo-Mpro) and complexed with molecule **1** were analyzed for the final outcome of root mean square deviation (RMSD), root mean square fluctuation (RMSF), and number of hydrogen bonds formation derived from simulation. Binding energies of the complexes were calculated by using MMGBSA [42] for every 1 ns trajectories, till 20 ns and the average binding energies with standard deviations were measured for accurate binding approximation and stability described elsewhere [49].

## 5. Conclusions

In the present study, the repositioning approach of SARS-CoV inhibitors, alongside QSARs, QSAR-based virtual screening, and molecular docking–molecular dynamics analyses were performed to identify new potent inhibitor candidates against main protease of SARS-CoV-2. A five parametric GA–MLR QSAR model was developed to identify the main pharmacophoric features that govern the Mpro inhibitory activity. Internal and external validation and other stringent tests according to OECD principles were performed for the developed model. From the present analysis, pharmacophoric features like non-ring Carbons and Nitrogens, amide Nitrogen, sp2-hybridized Carbons, lipophilic atoms, etc., appeared as prominent features that govern the Mpro inhibitory activity. The developed QSAR model possesses high external predictive ability and robustness for fitting and internal validation. In addition, virtual screening successfully offered new derivatives of molecule 1 and from food database with improved Ki values in the range from 0.59 to 11.59 nM. Additionally, molecular docking of active candidate **1** within the active pocket of Mpro shed the light about the important pharmacophoric moieties involved in the binding interactions which are responsible for the inhibitory potential. It appeared that molecule **1** occupied the S1 and S2 pockets of the active site. Molecular docking and MD analysis identified the crucial role of triazole and benzene rings in establishing lipophilic and H-bonding with the important residues like Leu141, Met165, Glu166, Asp187, Gln189, etc., of the active site Mpro. QSAR and molecular docking provided consensus, as well as complementary pharmacophoric features, which therefore have to be retained in developing potent and selective SARS-CoV-2 inhibitors. Lastly, the significantly high binding energy of compound 1 with Mpro vindicates the higher affinity and opens the new arena for a novel inhibitor drug against SARS-CoV-2. The results could be highly useful to develop a therapeutic agent for SARS-CoV-2.

## Figures and Tables

**Table 1:** Predicted (Pred) pKi and Ki for different variants of molecule 1 (#1 to 10) and food compounds (#11 to 20) used for QSAR-based virtual screening.

SN	Pred-pKi (M)	Pred-Ki (nM)
S341	9.226	0.594
S337	9.121	0.757
S342	9.121	0.757
S338	9.086	0.82
S339	8.842	1.439
S340	8.807	1.56
S293	8.499	3.17
S161	8.464	3.436
S251	8.464	3.436
S317	8.464	3.436
FoodS8291	8.46	3.467
FoodS6189	8.355	4.416
FoodS677	8.32	4.786
FoodS3568	8.32	4.786
FoodS4426	8.251	5.61
FoodS6919	8.251	5.61
FoodS4135	8.181	6.592
FoodS7495	8.181	6.592
FoodS1368	7.971	10.691
FoodS4841	7.936	11.588

**Table 2:** Docking score for top ten active molecules from the selected dataset of 62 molecules.

SN	Structure	Docking Score (kcal/mol)
1	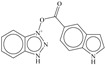	−5.997
2	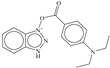	−7.008
3	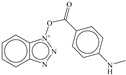	−6.42
4	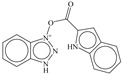	−5.886
5	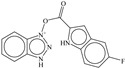	−6.041
6	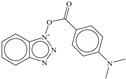	−6.48
7	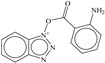	−5.866
8	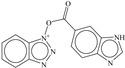	−6.331
9	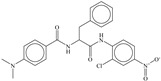	−7.774
10	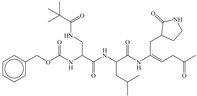	−9.931

**Table 3:** Structures and docking score for molecules having higher docking score in the present dataset.

SN	Structure	Docking Score (kcal/mol)
18	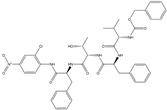	−10.285
60	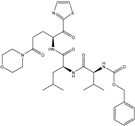	−10.259
19	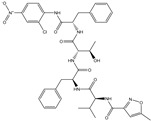	−10.159
21	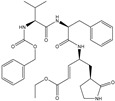	−10.026
57	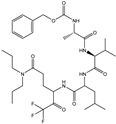	−9.975
10	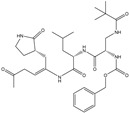	−9.931

**Table 4:** Docking scores along with the interacting amino acids for the least and most active molecules **1** and **62**.

SN	List of Interacting Amino Acids	Docking Score
1	His41, Met49, Tyr54, Phe140, Leu141, Asn142, Ser144, Cys145, His163, His164, Met165, Glu166 (strong H-bond), Asp187 (strong H-bond), Arg188, Gln189	−10.285
62	His41, Met49, Tyr54, Phe140, Leu141, Asn142, Ser144, Cys145, His163, His164, Met165 (weak H-bond), Glu166, Pro168, Val186, Asp187, Arg188, Gln189 (weak H-bond)	−9.358

**Table 5:** Simplified Molecular−Input Line−Entry System (SMILES) notations, Ki(nM), and pKi(M) for top five most active and least active molecules.

SN	SMILES	Ki (nM)	pKi (M)
1	c1cccc(c12)n(nn2)OC(=O)c(c3)ccc(c34)[nH]cc4	7.5	8.125
2	c1cccc(c12)n(nn2)OC(=O)c3ccc(cc3)N(CC)CC	11.1	7.955
3	CNc(cc1)ccc1C(=O)On(nn2)c(c23)cccc3	12.1	7.917
4	c1cccc(c12)n(nn2)OC(=O)c(c3)[nH]c(c34)cccc4	12.3	7.91
5	c1cccc(c12)n(nn2)OC(=O)c(c3)[nH]c(c34)ccc(F)c4	13.8	7.86
58	CCCN(CCC)C(=O)CC[C@@H](C(=O)C(F)(F)F)NC(=O)[C@H](CC(C)C)NC(=O)[C@H](C(C)C)NC(=O)OCc1ccccc1	363,000	3.44
59	CCN(CC)C(=O)CC[C@@H](C(=O)c1nccs1)NC(=O)[C@H](CC(C)C)NC(=O)OCc2ccccc2	462,000	3.335
60	C1COCCN1C(=O)CC[C@@H](C(=O)c2nccs2)NC(=O)[C@H](CC(C)C)NC(=O)[C@H](C(C)C)NC(=O)OCc3ccccc3	478,000	3.321
61	CCCN(CCC)C(=O)CC[C@@H](C(=O)C(F)(F)F)NC(=O)[C@H](CC(C)C)NC(=O)OCc1ccccc1	584,000	3.234
62	CCN(CC)C(=O)CC[C@@H](C(=O)c1nccs1)NC(=O)[C@H](C(C)C)NC(=O)OCc2ccccc2	614,000	3.212

SN = serial number.

## Data Availability

The data is available in the supplementary section.

## Supplementary Materials

The following are available online at https://www.mdpi.com/article/10.3390/ph14040357/s1 Table S1: SMILES notations, Ki, pKi, and values of all molecular descriptors used in the model. Table S2: Details regarding performance of model. Table S3: Correlation matrix for molecular descriptors present in the developed QSAR model, statistical parameters for used for validation of QSAR models and the formulae for the statistical parameters. Table S4: SMILES notations, pKi, Ki, and values of molecular descriptors for molecules used for QSAR-based virtual screening. Figure S1: Different graphs associated with model (a) graph of experimental vs. residual values (b) Y-scrambling plot.

## Abbreviations

SMILES	Simplified Molecular-Input Line-Entry System
GA	Genetic Algorithm
MLR	Multiple Linear Regression
QSAR	Quantitative Structure−Activity Relationship
WHO	World Health Organization
ADMET	Absorption, Distribution, Metabolism, Excretion, and Toxicity
OLS	Ordinary least square
SARS-CoV	Severe Acute Respiratory Syndrome Coronavirus
QSARINS	QSAR Insubria
HTS	High-throughput screening
MDS	Molecular Dynamics Simulation
Mpro	Main protease
OECD	Organisation for Economic Co-operation and Development
